# The dataset for the inflammatory response during experimental infection and treatment of dogs with *Babesia rossi*

**DOI:** 10.1016/j.dib.2022.108475

**Published:** 2022-07-19

**Authors:** Brogan Kim Atkinson, Peter Thompson, Estee Van Zyl, Amelia Goddard, Yolandi Rautenbach, Johan Petrus Schoeman, Varaidzo Mukorera, Andrew Leisewitz

**Affiliations:** a Department of Companion Animal Clinical Studies, Faculty of Veterinary Science, University of Pretoria, Pretoria, South Africa; b Department of Production Animal Studies, Faculty of Veterinary Science, University of Pretoria, Pretoria, South Africa

**Keywords:** Cytokines, C-reactive protein, Leukocytes, Treatment, Parasitemia, Anaemia*.*

## Abstract

*Babesia rossi* causes severe morbidity and mortality in dogs in sub-Saharan Africa. This was an experimental study designed to observe systemic changes caused by *Babesia rossi* infection within a canine disease model as well as investigate the influence of inoculum dose and treatment on the progression of inflammation and clinical disease. Six healthy male beagle dogs formed the study population, one dog was splenectomised and used to raise the infectious inoculum, three were administered a high *B. rossi* infectious dose and two a low infectious dose. Clinical examination, complete blood count (CBC) and C-reactive protein (CRP) were determined daily. Cytokines were quantified on stored plasma collected during the study, using a canine specific cytokine magnetic bead panel (Milliplex©). The experiment was terminated, and treatment administered once predetermined experimental or humane endpoints were reached. The data and information provided in the following article is the summary of all data points collected over the course of the eight-day experimental infection.

## Specifications Table

**Table:** 

Subject	Veterinary science and medicine Parasitology
Specific subject area	This was an experimental study during which beagle dogs were inoculated with a virulent canine parasite, *Babesia rossi,* and the progression of the disease and inflammation were evaluated over time. The dogs were then treated, and the influence of treatment was investigated [1].
Type of data	Table: There is one table in this article providing the values for each variable within the groups and the statistical difference between the groups as well as each group compared to baseline data [1].
How the data were acquired	Venous samples for CBC were collected atraumatically into EDTA Vacutainer Brand Tubes (Beckton Dickinson Vacutainer Systems, UK) from the jugular and were analysed using the ADVIA 2120 (Siemens, Munich, Germany). Serum biochemistry samples were collected in Serum Vacutainer Brand Tubes (Beckton Dickinson Vacutainer Systems, UK), and the CRP (using canine specific immunoturbidimetric CRP method, Gentian, Norway) were analysed using the Cobas Integra 400 plus (Roche, Switzerland). On completion of the experimental period, batched EDTA plasma samples were thawed at room temperature and used to determine granulocyte-macrophage colony-stimulating factor (GM-CSF), interferon gamma (INFγ), interleukin 2 (IL-2), IL-6, IL-7, IL-8, IL-15, IL-10, IL-18, TNF-a, INFγ-induced protein 10 (IP-10), keratinocyte chemoattractant (KC-like) and Monocyte chemoattractant protein-1. Concentrations were determined in duplicate by fluorescent-coded magnetic beads (MagPlex-C; MILLIPLEX. MAP Kit, Canine Cytokine Magnetic Bead Panel, 96-Well Plate Assay, CCYTO-90K, Millipore, Billerica, MA), based on the Luminex xMAP technology (Luminex 200, Luminex Corporation, Austin, TX). Two quality controls were included in the plate as internal quality controls. The assay was performed according to the manufacturer's instructions. Cytokine concentrations were determined by comparing the optical density of the samples to the standard curves, produced from standards run on the same plate. The minimum detectable concentrations of the cytokines provided by the manufacturer were regarded as the detection limits in this study. Measurable values below the detection limit were assigned a value equal to the minimum detectable concentration for the respective cytokine and those with no measurable values were set as zero [1]. Blood pressure was measured using the Vet HDO® MDPro and the HDO management software (S + B medVET GmbH, Germany) [2], [3]. The following data was collected every day for all the dogs in the study until endpoints were reached and the dogs were treated: Habitus and mental status (scored 1+ - 4+): Habitus: 1+ Lethargic and non-responsive; 2+ Lethargic but responsive; 3+ Alert and responsive; 4+ Bright alert and responsive; Appetite (scored 1+ - 4+): Appetite: 1+ Refusal to eat; 2+ Eats when hand or syringe fed; 3+ Eating unassisted but inadequate intake; 4+ Eating very well; Temperature; Pulse (rate, rhythm, and quality); Respiratory rate and type; Mucous membrane colour and capillary refill time; Thoracic auscultation; Abdominal palpation; Hydration status; Blood smear examination; Blood pressure.
Data format	Raw: https://data.mendeley.com/datasets/zyhy8z69kt/1 Analysed: This is included in the supplementary data.
Description of data collection	An automated haematology was performed within 1 hour of blood collection. The remaining volume of blood was centrifuged and aliquoted within 30 minutes of collection for storage of EDTA plasma at -80⁰C for batching and running of the cytokines on completion of the study period. Serum samples were allowed to clot for 10 minutes, and were analysed within 30 minutes of collection.
	Blood pressure was measured daily. The dogs were were conditioned for several weeks leading up to the study period, to minimize procedure associated stress [1]. Inclusion criteria for experimentally infected dogs: Clinically healthy, based on clinical variables (temperature, pulse, respiration rate, abdominal palpation, capillary refill time and mucous membrane colour, haematology and biochemistry) remaining within normal limits [1]. They were free from any regional blood-borne parasitic infections (Confirmed by PCR-RLB prior to experimental infection), including *Babesia rossi*. All vaccinations and deworming were current [1]. Exclusion criteria for experimentally infected dogs: Co-infection with other blood parasitic infections [1]. The presence of comorbid disease [1]. The splenectomised dog was excluded from the data collection [1].
Data source location	• Institution: University of Pretoria, Faculty of Veterinary Science • City/Town/Region: Onderstepoort, Pretoria • Country: South Africa
Data accessibility	Repository name: Mendeley Data and Digital Commons Data Data identification number: 10.17632/zyhy8z69kt.1 Direct URL to data: https://data.mendeley.com/datasets/zyhy8z69kt/1
Related research article	B.K. Atkinson*, P. Thompson, E. Van Zyl, A. Goddard, Y. Rautenbach, J.P. Schoeman, V. Mukorera, A. Leisewitz, Kinetics of the inflammatory response during experimental Babesia rossi infection of beagle dogs, J. Vet. Parasitology, In Press.

## Value of the Data

• This time course study of markers of inflammation in experimental *Babesia rossi* infection will improve our understanding of the evolution of the systemic inflammatory response associated with canine babesiosis [4], [5], [6], [7], [8], [9], [10], [11], [12], [13]. This data will add to the growing database for Babesia research and will assist with our ongoing endeavours to optimise our approach to the treatment of this disease. We will compare this canine disease with other important blood-borne protozoal infections affecting humans and animals, to better grasp the pathophysiology of this group of infections [4], [5], [6], [14], [15], [16], [17]. In addition to this, an established experimental *B. rossi* infection may potentially provide a disease model for blood-borne protozoal disease and haemolytic disease [14], [17], [18]. This study provides the groundwork for larger scale and more in-depth investigations.
• The information gained from this study benefits veterinarians faced with the treatment of this disease, particularly in Sub-Saharan Africa [19]. It may also benefit veterinarians worldwide by improving our understanding of the physiological reaction to this group of infectious agents and the impact treatment has on the progression of disease [4], [5], [6]. Using this infection as a model for similar infectious diseases in humans, such as malaria and zoonotic babesiosis, as well as a model for haemolytic disease could provide greater understanding of similar diseases and provide future treatment targets benefiting both human and veterinary health care [14], [15], [16], [17]. Finally, the greater research community, particularly those involved in infectious diseases and systemic inflammation would benefit from the information gained by comparing this disease model with changes seen in similar pathogens.
• The data from this experimental infection may be compared to changes seen in natural infections, providing more information on the influence of this parasite on canine inflammatory pathophysiology [1], [7], [8], [10], [11], [12]. Differences between this experimental infection and natural infections can be identified, allowing the refinement of future studies. There are several experimental studies on the inflammatory response to different canine *Babesia* species and our study will provides the groundwork for comparisons between the various species and may highlight possible reasons for the increased virulence of this particular species in the canine population [4], [5].

## 1 Data Description

Table 1: Cytokine concentrations during B. rossi infection and or 4 days after treatment.

* Significant difference when applying linear mixed models, P<0.05. IFNγ (interferon gamma), KC-like (keratinocyte chemoattractant), MCP-1 (Monocyte chemoattractant protein 1), IL (Interleukin), GM-CSF (granulocyte-macrophage colony-stimulating factor), TNFα (tumor necrosis factor alpha) and IP (interferon gamma-induced protein).

Supplementary data: This table contains the range of each parameter measured during the study with the statistical significance when comparing the groups between each other and between each group and the baseline.

Raw data: The raw data collected for every variable over the course of the study, for independent evaluation.

## 2 Experimental Design, Materials and Methods

### 2.1 Study design

This was a prospective longitudinal observational study that included six purpose bred sterilised male beagle dogs [1]. One dog was splenectomised and used to raise a viable parasite inoculum from cryopreserved wild type *Babesia rossi* [1]. The remaining five dogs were experimentally infected with either a high or low dose *Babesia rossi* parasite inoculum [1]. Samples collected from the five dogs that underwent the experimental infection provided the baseline data to which the changes during infection were compared [1]. The samples were collected on two separate occasions two weeks apart, prior to the onset of the study period [1].

### 2.2 Study setting

The beagles were housed at the University of Pretoria Biomedical Research Centre (UPBRC) from eight weeks of age until the end of the experimental period. The remaining five beagles were rehomed as pets. All clinical examinations as well as sample collection were performed at the UPBRC. The complete blood count and biochemistry analysis was performed at Clinical Pathology Laboratory of the Onderstepoort Veterinary Academic Hospital and the cytokine analysis was performed in the laboratory of the Department of Veterinary Tropical Diseases.

### 2.3 Study population

There were six purpose bred intact male beagle dogs. All dogs were permanently identified by microchip implantation (Back Home®, Virbac, South Africa).

Inclusion criteria for splenectomised dog [1]:

• The dog was clinically healthy, clinical variables (temperature, pulse, respiration rate, abdominal palpation, capillary refill time and mucous membrane colour, haematology and biochemistry) were within normal limits.
• The dog was free from *Babesia rossi* or any other regional blood-borne parasitic infections (Confirmed by polymerase chain reaction and reverse line blotting (PCR-RLB) prior to experimental infection).
• All vaccinations and deworming were current.

Exclusion criteria for dog to be splenectomised [1]:

• Co-infection with other blood parasitic infections namely *Theileria sp., Anaplasma sp.* and *Ehrlichia sp.* (Evaluated by PCR prior to experimental infection).
• The presence of comorbid disease.

Inclusion criteria for experimentally infected dogs [1]:

• They were clinically healthy, clinical variables (temperature, pulse, respiration rate, abdominal palpation, capillary refill time and mucous membrane colour, haematology and biochemistry) were within normal limits.
• They were free from *Babesia rossi* or any other regional blood-borne parasitic infections (Confirmed by PCR-RLB prior to experimental infection).
• All vaccinations and deworming were current.

Exclusion criteria for experimentally infected dogs [1]:

• Co-infection with other blood parasitic infections (Evaluated by PCR prior to experimental infection).
• The presence of comorbid disease.
• The splenectomised dog was excluded from the data collection.

### 2.4 Sample method

The five dogs meeting the necessary inclusion criteria were included in the study and randomly assigned to either a low infectious dose (LD) group or high infectious dose (HD) [1].

### 2.5 Sample size

The five spleen-whole dogs were subject to sample collection for baseline control data [1].

These dogs were then randomly divided into 2 groups [1]:

LD group:

• Dogs experimentally infected with a low dose (10^4^ infected red blood cells) of *Babesia rossi* parasite inoculum
• Two dogs were allocated to this group.

HD group:

• Dogs experimentally infected with a high dose (10^8^ infected red blood cells) of *Babesia rossi* parasite inoculum
• Three dogs were allocated to this group.

The high and low infectious doses were based on doses previously described in an experimental *B. canis* infection [1]. Higher doses were selected to account for possible parasite loss during dilution preparation and to improve the chances of establishing a viable infection in the current experimental model [1].

**Fig. 1:**
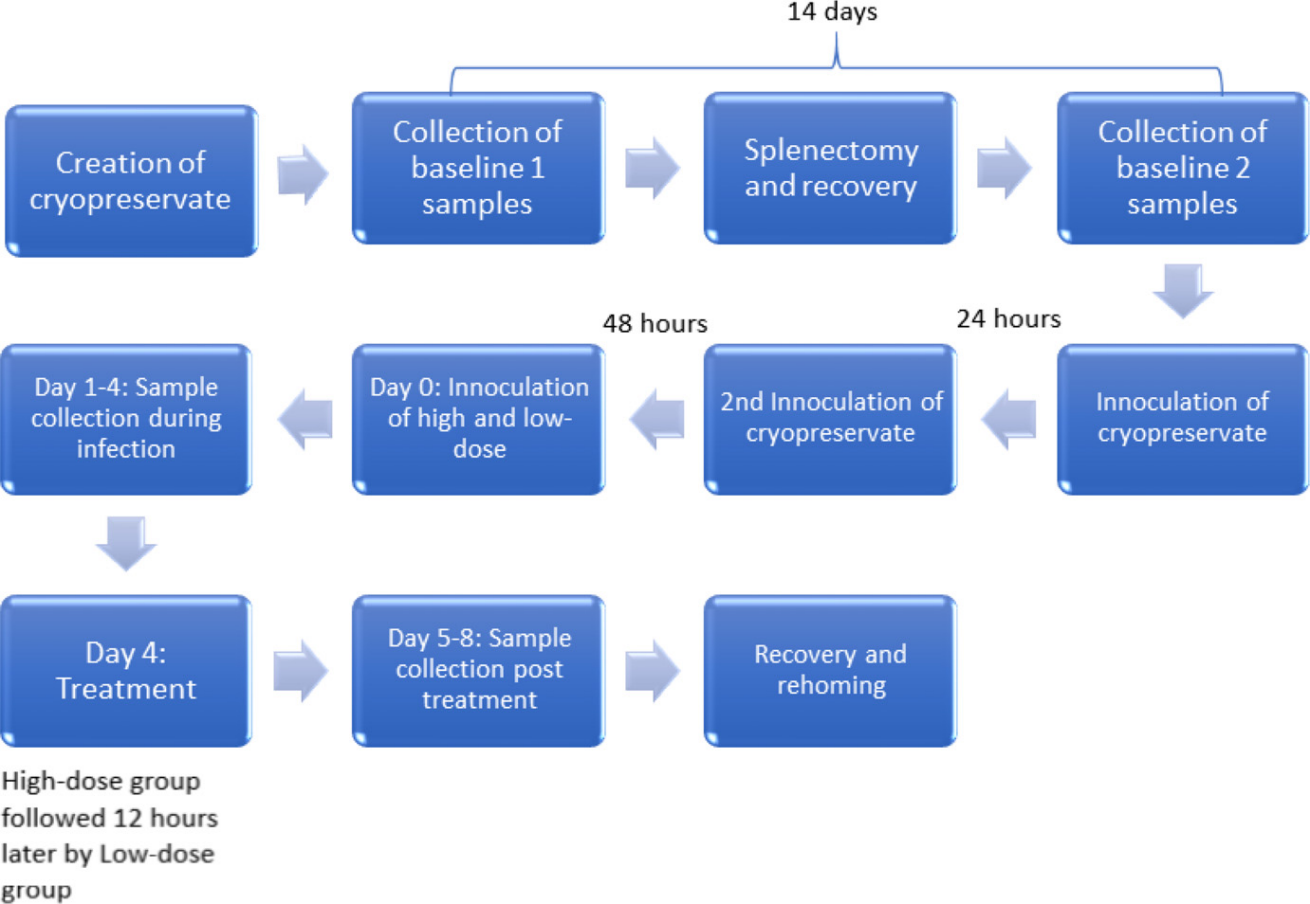
A flow diagram of the experimental period [1].

## 3 Experimental design

### 3.1 Phase 1

Creation of a pure wild-type *Babesia rossi* parasite cryopreservate from a naturally infected dog presented to the Outpatients clinic of the Onderstepoort Veterinary Academic Hospital for veterinary care. The mono-infection and confirmation of the speciation of the parasite was determined by PCR-RLB [1].

#### 3.1.1 Creation of *B. rossi* cryopreservate [1]

Equipment, materials, and facilities:

• Blood infected with parasites
• Dimethylsulphoxide (DMSO)
• Heparin Vacutainer Brand Tubes (Beckton Dickinson Vacutainer Systems, UK)
• Sterile 2 mL or 4 mL cryotubes (Corning incorporated, USA) and labels for cryotubes
• Ice and racks to contain tubes
• 50 mL Erlenmeyer flask
• Pipettes (including micropipettes) and pipette tips
• Syringes
• Microscope slides
• Cover slips (24 × 24 mm)
• -80 ⁰C deep freezers

#### 3.1.2 Procedure

Blood was collected in EDTA Vacutainer Brand Tubes (Beckton Dickinson Vacutainer Systems, UK) from a *B. rossi* infected dog. The blood had a parasitaemia of approximately 10 %. The blood was stored at 4⁰C for 2 hours prior to further processing. A 10% DMSO and blood mixture was made using 8 mL of blood transferred from the collection tubes into a 50 mL flask and kept on melting ice. Slowly 0.88 mL of DMSO was added drop by drop, at a rate of about 10 seconds between drops, using a 1 mL pipette, while gently swirling the blood in the flask (whilst keeping the flask in melting ice water). The contents were gently mixed by pipetting them up and down several times. The blood was dispensed into 2 mL cryotubes, kept in a rack on the laboratory bench, also in melting ice water. The cryotubes were then shortly transferred to and stored in a -80⁰C freezer.

Just prior to infection of the splenectomised dog, the stored samples were thawed in a water bath adjusted to 37⁰C and kept under water until just thawed. The contents were transferred to a syringe and inoculated intravenously within 15 minutes.

### 3.2 Phase 2: Determination of baseline data and creation of infectious inoculum [1]

All five spleen-whole dogs had their clinical variables evaluated and recorded. Blood was collected using a vacutainer system into a serum and EDTA tube for the determination of cytokine concentrations, albumin concentration, C-reactive protein concentration and a complete blood count (for neutrophil and monocyte counts). A small amount of fresh whole blood was used directly from the needle for the determination of the glucose concentrations using a handheld reader (specified later). Blood was collected directly into a heparinized syringe for the determination of venous blood gas – lactate measurement. All data and sample collection were performed once daily between 8 and 10 am prior to feeding.

The randomly selected dog was splenectomised, by a specialist surgeon employed at the Onderstepoort Veterinary Academic Hospital (OVAH) and conducted in the UPBRC theatre. Once completely recovered (two weeks post-operatively), the splenectomised dog was inoculated with 2 ml of cryopreserved wild-type infected blood. A second dose was repeated 24 hours later.

The parasitaemia of the splenectomised dog was determined manually twice daily on central venous blood, starting 12 hours after the initial inoculum dose.

The parasitaemia throughout the study was determined as follows [1], [20]:

• Blood smears were stained with Kyro-quick (Kyron Laboratories, Benrose, South Africa), a Romanowski stain, and scored at 1000 times magnification with the aid of a digital image analysis program (Optimas 6 for Win 95/ NT 4.0, Media Cybernetics, distributed by Carl Zeiss Ltd., Randburg, South Africa).
• Multiple digital photographs of 3 sections namely: the red cell area, the feather edge and the sides (straight lateral margins) of the smears were transferred to a computer screen and magnified. The images were saved and printed out. The red blood cells were counted using markers over counted cells to ensure accurate counting.
• Free parasites were not counted.
• Unparasitized RBC and parasitized RBC were counted in each field, with a different marker for parasitized vs unparasitized.
• At least 650 RBC were counted in each of three fields. A total of at least 1950 RBC were examined per smear. Each oil immersion field was completely counted to avoid artificially increased parasitaemia had only portions been examined.
• The counting and scoring of all slides were done by the author and the slides were archived.
• The results were expressed as percent parasitized red blood cells.
• A score of <0.05% was given if no parasitized RBC were detected in the designated areas but were observed somewhere else on the smear.
• A score of 0% was assigned if no parasitized RBC were detected on the smear after 15 minutes of scanning the slide without counting.

Once a parasitaemia above >0.05% was identified, citrated whole blood was collected from the splenectomised dog and the parasitaemia was calculated. When the percentage parasitaemia was calculated, it was multiplied by the RBC count obtained from a complete blood count analysis.

• Number of parasitized RBC (per mL) = % parasitized RBC X RBC count (per mL)

Dilutions to obtain desired inoculum doses were done as follows:

• A culture medium was utilised for the dilution of the parasitised blood:
  ○ Culture Media RPMI 1640 (ThermoFischer Scientific, USA)
  ○ Filtered water (1 L)
  ○ 25 millimolar Hepes
  ○ NaHCO_3_ (Sodium bicarbonate) 2.1 g/L
  ○ Sodium pyruvate (1 millimolar/L)
  ○ Gentamycin (100 mg/mL)

The RPMI (powder form) was dissolved in 600 mL of ultra-pure water. 2.1 g of NaHCO3 was added to the above suspension. The pH was adjusted to 7.3, using NaOH 5M. 10 mL of sodium pyruvate was then added, in sterile conditions to avoid contamination of the stock solution. Ultra-pure water was added up to 1 Litre and the solution was filtered, using 0.22 µm pore filter units and a peristaltic pump. Gentamicin 10 mg/L was added after filtration. The solution was aliquoted into adequate volumes and stored at 4⁰C.

• Desired inoculum doses were 10^4^ and 10^8^ parasitized RBC, for the low dose (LD) and high dose (HD) groups respectively.
• The dilution was done as follows:
  ○ 17mL of blood was collected from the splenectomised donor dog into 3mL Citrate Phosphate Dextrose Adenine (CPDA-1) anticoagulant, with a final concentration of 0.15 ml of CPDA-1 per ml of blood admixture.
  ○ An additional 1 mL of blood was collected separately directly into and EDTA Vacutainer Brand Tubes (Beckton Dickinson Vacutainer Systems, UK) for CBC determination.
  ○ Percent parasitaemia on day of blood collection:
    ■ 33 parasitized red blood cells/1950 red blood cells counted = 1.69 % parasitaemia
• Percent parasitaemia (in decimal form) x red blood cell count (determined from CBC)

0.0169 x (6.15 × 10^9^/mL) = 0.1041 × 10^9^ parasitized red blood cells/mL x 10
= 1.041 × 10^8^ parasitized red blood cells/mL.

• To account for citrate dilution:

(1.041 × 10^8^ parasitized red blood cells/L x 17 mL of blood)/ 20mL total fluid volume
= 0.88465 × 10^8^ parasitized red blood cells/mL.

• To get a high dose inoculum of 10^8^ parasitized red blood cells:

1 × 10^8^ parasitized red blood cells/ (0.88465 × 10^8^ parasitized red blood cells/mL)
= 1.14 mL of total Citrated blood provided the high dose of 10^8^ parasitized red blood cells.

• The low dose was achieved with serial dilutions as follow:
• Three serial dilutions of:
  ○ 1 mL of parasitized blood (Infectious dose of 8.8465 × 10^7^/mL) with 9 mL diluent
  ○ 1 mL of parasitized blood (Infectious dose of 8.8465 × 10^6^/mL) with 9 mL diluent
  ○ 1 mL of parasitized blood (Infectious dose of 8.8465 × 10^5^/mL) with 9 mL diluent
• Then a serial final dilution of:
  ○ 1 mL of parasitized blood (Infectious dose of 8.8465 × 10^4^/mL) with 7.8465 mL diluent.
• This provided a final low dose concentration of 10^4^ parasitized red blood cells/mL.
• Before the low dose infectious inoculum was given, 1 mL of the sample was centrifuged, and a smear of the pellet was used to confirm presence of parasitized RBC. Once parasitaemia was confirmed 1mL of this solution was used as the low dose.

### 3.3 Phase 3: Infection of the experimental dogs [1]

The splenectomised dog was drug cured with diminazene aceturate (3.5mg/kg SC dosed once) straight after blood collection for the experimental infections.

Infection of the experimental dogs with fresh whole blood diluted to achieve the two parasite doses required, namely 10^4^ and 10^8^ parasitized RBC. The dogs were inoculated as follows:

• LD group (2 dogs): 1 mL containing a total of 10^4^ *Babesia rossi* parasitized RBC was given intravenously.
• HD group (3 dogs): 1.14 mL containing a total of 10^8^ *Babesia rossi* parasitized RBC was given intravenously.

Blood was collected once daily between 8 and 10 am from all 5 dogs for the duration of the experiment (see Table 1). Following the unexpected death of one dog in the HD group on day 4, the remaining HD group dogs were sampled and treated. For concerns over the wellbeing of the remaining LD group dogs, they were sampled 12 hours later (for the second time that day) and then treated. All the dogs were drug cured with diminazene aceturate at 3.5mg/kg subcutaneously once off and received additional supportive therapy as needed (including blood transfusions).  The remaining dogs (including the splenectomised dog) recovered well and were rehomed as pets following a minimum of 2 weeks of recovery time

#### 3.3.1 The experimental endpoints (any one criterium determined the point of treatment) [1]

Humane endpoints:

• A haematocrit (packed cell volume) of between 10 and 15%.
• A dog with a habitus score of 1+.
• A dog showing neurological signs (such as seizure activity) whether due to neuroglycopenia or not.
• A dog with clinical evidence of lung pathology in which there is arterial blood gas evidence of acute respiratory distress syndrome (arterial pO_2_<60mmHg, normal > 80mmHg at our altitude).
• A dog with a serum creatinine > 200mmol/L (normal <140 mmol/L).
• A dog that demonstrates haemoconcentration (defined as a PCV >55%) in the face of obvious haemolysis (macroscopically evident haemoglobinuria and or haemoglobinaemia).

Experimental endpoint:

• Any dog that lives to 20 days post infection will be treated on the 20^th^ day post infection.

The HD group was treated on day four in the morning (at 96 hours). The LD group was treated 12 hours later (at 108 hours) despite not having reached the same degree of disease severity as the HD group.

### 3.4 Experimental procedures

#### 3.4.1 Sample collection schedule and volumes [1]

All sample collection and blood smear evaluation was performed by the main investigator and assisted by supervisors and co-workers. Clinical examinations and all blood samples were collected from the jugular vein with 21G vacutainer needles (Precision Glide^TM^, UK).

For this experiment the blood collection schedule was as follows:

**Table 1:** Sample collection schedule with the details of what data was collected of different days.

Parameter to be measured	Schedule	Test to be run
Habitus, appetite, temperature, pulse, respiratory rate, mucous membrane colour, blood pressure	Daily on all dogs	General health status and wellbeing of all dogs.

Blood smear	Daily on small volume of peripheral blood from the ears of all dogs and from the EDTA sample below.	Parasitaemia determination.
0.5 mL of whole blood in EDTA tube from jugular collection	Daily for CBC from all dogs from day 0 until endpoints were reached and infected dogs were treated.	Complete blood count and cytokine concentration determination.

3 mL of whole blood in serum tube from jugular collection	Every second day from all dogs from day 0 (i.e., day 0 and then 2/4/6 etc) until endpoints were reached and infected dogs were treated.	Biochemistry: Total serum protein, glucose, albumin, C-reactive protein.

0.5 mL of whole blood in heparinized syringe from jugular collection	Every second day from all dogs from day 0 (i.e., day 0 and then 2/4/6 etc) until endpoints were reached and infected dogs were treated.	Venous blood gas and lactate.

Small amount of fresh whole blood remaining in the needle after collection.	Daily on all dogs.	Lactate and glucose from handheld readers.

Haematology: Venous samples for CBC were collected atraumatically into EDTA Vacutainer Brand Tubes (Beckton Dickinson Vacutainer Systems, UK) from the jugular and were run on an ADVIA 2120 (Siemens, Munich, Germany). A differential count was performed manually by an experienced laboratory technologist. An automated haematology was performed (ADVIA 2120i, Siemens, Germany) within 1 hour of blood collection. The remaining volume of blood was then centrifuged and aliquoted within 30 minutes of collection for storage of EDTA plasma at -80⁰C.

Serum biochemistry samples were collected in Serum Vacutainer Brand Tubes (Beckton Dickinson Vacutainer Systems, UK), and the CRP (using canine specific immunoturbidimetric CRP method^h^, Gentian, Norway) [10], albumin (using a colorimetric assay with bromocresal green, Roche Diagnostics, Switzerland) were determined. This sample was allowed to clot for 10 minutes, samples were run, and the remaining serum volumes were aliquoted within 30 minutes of collection for storage at -80°C.

Glucose was analysed using fresh whole blood with the point of care AlphaTRAK 2 (Zoetis, USA).

Blood gas samples were collected anaerobically into a commercially prepared heparinized syringe (BD A-Line, arterial blood collection syringe, Becton, Dickinson and Company, UK) using a 21G needle from the jugular. Lactate was determined from the venous blood gas analysis, analysed within 20 minutes (Rapidpoint 405, Siemens).

At the conclusion of the study, the stored plasma samples were thawed at room temperature and used to analyse GM-CSF, IFN-y, IL-2, IL-6, IL-7, IL-8, IL-15, IL-10, IL-18, TNF-a, IP-10, KC-like and MCP-1 concentrations. These were analyzed using fluorescent-coded magnetic beads (MagPlex-C; MILLIPLEX. MAP Kit, Canine Cytokine Magnetic Bead Panel, 96-Well Plate Assay, CCYTO-90K, Millipore, Billerica, MA), based on the Luminex xMAP technology (Luminex 200, Luminex Corporation, Austin, TX).

All serum and plasma not utilized remains stored at -80⁰C and is logged in the OVAH biobank.

Blood pressure was measured using the Vet HDO® MDPro and the HDO management software (S + B medVET GmbH, Germany). The protocol for blood pressure measurement was standardised as follows [3]:

• The environment was isolated, quiet, and away from other animals. The same environment was used every day and the dogs were conditioned for 4 weeks prior to the onset of the experiment.
• No sedation was used, and the dogs were allowed 5-10 minutes to relax and become accustomed to the environment.
• Each dog was gently restrained in right lateral recumbency.
• The cuff width was approximately 40% of the circumference of the cuff site and the cuff size was recorded at every measurement.
• The cuff was placed around the tail base.
• All blood pressure measurements were performed by the same person every day.
• The dogs were calm and relatively motionless as they were all well-conditioned to the process prior to the start of the experimental period.
• The first measurement was discarded. Five consecutive and consistent (<20% variability in systolic values) were recorded and the average of the values was calculated to obtain the blood pressure measurement.
• Written records were kept daily of the readings.

### 3.5 Observational/analytical procedures [1]

The following data was collected every day for all the dogs in the study until endpoints were reached and the dogs were treated:

• Habitus and mental status (scored 1+ - 4+)
  ○ Habitus: 1+ Lethargic and non-responsive; 2+ Lethargic but responsive; 3+ Alert and responsive; 4+ Bright alert and responsive
• Appetite (scored 1+ - 4+)
  ○ Appetite: 1+ Refusal to eat; 2+ Eats when hand or syringe fed; 3+ Eating unassisted but inadequate intake; 4+ Eating very well
• Temperature
• Pulse (rate, rhythm, and quality)
• Respiratory rate and type
• Mucous membrane colour and capillary refill time
• Thoracic auscultation
• Abdominal palpation
• Hydration status
• Blood smear examination
• Blood pressure
• Haematology (CBC)
• EDTA plasma was stored for cytokine determination at the end of the experiment
• Blood glucose

A data collection sheet was drawn up to record this daily information.

The following was only collected every second day for all dogs:

• A serum sample for biochemistry (Albumin and C-reactive protein)
• Venous blood gas

### 3.6 Cytokine Analysis [1]

The stored plasma samples were thawed at room temperature and used to determine GM-CSF, IFN-y, IL-2, IL-6, IL-7, IL-8, IL-15, IL-10, IL-18, TNF-a, IP 10, KC and MCP-1 concentrations. These cytokines were analyzed in duplicate by fluorescent-coded magnetic beads (MILLIPLEX® MAP Kit, Canine Cytokine Magnetic Bead Panel, 96-Well Plate Assay, CCYTO-90K, Millipore, Billerica, MA, USA), based on the xMAP® technology (Bio-Plex®-MAGPIX™ 200, Bio-Rad Laboratories, Inc., California, USA). Two quality controls were included in the plate as internal quality controls. The assay was performed according to the manufacturer's instructions.

Preparation of the plasma samples:

• EDTA plasma samples were utilized. Samples were thawed, mixed well by vortexing and then centrifuged prior to use to remove any particulate material.

Preparation of reagents for immunoassay:

• The vial containing the premixed beads was sonicated for 30 seconds and then vortexed for 1 minute to ensure thorough mixing as contents prior to use.
• Quality control 1 and 2 were reconstituted with 250 µL of deionized water, inverted several times and vortexed. These were then allowed to sit for 10 minutes.
• The 10X Wash Buffer was brought to room temperature and mixed well. 60 mL of Wash Buffer was diluted with 540 mL of deionized water.
• 1 mL of deionized water was added to the lyophilized Serum Matrix, mixed well and given at least 10 minutes to reconstitute completely. 2 mL of Assay Buffer was added to this solution and mixed well.

Preparation of Canine Cytokine Panel Standards

• The Canine Cytokine Panel Standard was reconstituted with 250 µL of deionized water, inverted, vortexed for 10 seconds and then left to stand for 10 minutes before vortexing it a second time. This was Standard 7.
• 6 Polypropylene microfuge tubes were labelled Standard 1 to 6 respectively and 150 µL of Assay Buffer was added to each tube.  A four-fold dilution was then performed by adding 50 µL of Standard 7 to the Standard 6 tube and mixed thoroughly. This process was repeated by adding 50 µL of Standard 6 to the Standard 5 tube and so on until all 7 standards were prepared.

**Fig. 2:**
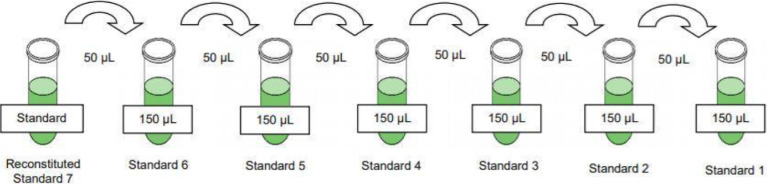
Standard preparation: The preparation of each Standard sample (1-7) using serial dilution of the reconstituted Standard with Assay Buffer, both provided by the manufacturer as part of the cytokine analysis kit.
• After the dilutions the tubes contained the following concentrations for constructing the standard curves:

**Table 2:** Standard concentrations: The concentrations of interferon gamma and the remaining twelve cytokines after the preparation of the Standard samples, these values were used the create the Standard curve against which the experimental samples were compared and concentrations were determined.

Standard tube number	IFN-γ (pg/mL)	All Other Analytes (pg/mL)
1	2.44	12.2
2	9.77	48.8
3	39.1	195
4	156	781
5	625	3125
6	2500	12500
7	10000	50000

The immunoassay procedure:

• All reagents were warmed to room temperature (20-25⁰C).
• A sample well map was created, please see appendix D for detailed map.
• The immunoassay procedure:

Sealed and placed in the plate shaker for 10 minutes at room temperature. Assay buffer was decanted, and residue removed. The plate was incubated overnight at 4⁰C on a plate shaker. Well contents were removed, and the plate was washed twice with Wash Buffer using an automatic plate washer (Bio-Plex Pro™ Wash Station, Laboratories, Inc., California, USA). The plate was sealed and incubated on a plate shaker for 1 hour at room temperature. Contents were not aspirated. The plate was sealed and incubated on a plate shaker for 30 minutes at room temperature. Well contents were removed, and the plate was washed twice with Wash Buffer using an automatic plate washer. All equipment was set to manufacturers specifications. A standard curve was constructed using the concentrations obtained in from the standards in the wells and sample concentrations were calculated accordingly. The minimum detectable concentrations of the cytokines provided by the manufacturer were regarded as the detection limits in this study and measurable values below the detection limit were assigned a value equal to the minimum detectable concentration for the respective cytokine and those with no measurable values were set as zero.

Sensitivity of the assay:

• Standard curve range for all the reagents: 12.2 – 50 000 pg/mL

**Table 3:** Immunoassay sensitivity: The minimum detectable concentrations for the cytokines analysed. Granulocyte-macrophage colony-stimulating factor (GM-CSF), interferon gamma (INFγ), interleukin 2 (IL-2), IL-6, IL-7, IL-8, IL-15, IL-10, IL-18, tumour necrosis factor alpha (TNF-α), INFγ-induced protein 10 (IP-10), keratinocyte chemoattractant (KC-like) and Monocyte chemoattractant protein-1.

Analyte	Minimum detectable concentrations
GM-CSF	9.2 pg/mL
IFNγ	13.6 pg/mL
IP-10	3.2 pg/mL
IL-2	3.5 pg/mL
IL-6	3.7 pg/mL
IL-7	7.5 pg/mL
IL-8	21.7 pg/mL
IL-10	8.5 pg/mL
IL-15	9.0 pg/mL
IL-18	5.8 pg/mL
KC-like	5.3 pg/mL
MCP-1	21.0 pg/mL
TNFα	6.1 pg/mL

Immunoassay precision:

**Table 4:** Immunoassay precision as provided by the manufacturer. Granulocyte-macrophage colony-stimulating factor (GM-CSF), interferon gamma (INFγ), interleukin 2 (IL-2), IL-6, IL-7, IL-8, IL-15, IL-10, IL-18, tumour necrosis factor alpha (TNF-α), INFγ-induced protein 10 (IP-10), keratinocyte chemoattractant (KC-like) and Monocyte chemoattractant protein-1. CV: coefficient of variation.

Analyte	Intra-Assay precision (CV%)	Inter-Assay precision (CV%)
GM-CSF	<5	<15
IFNγ	<5	<15
IP-10	<5	<15
KC-like	<5	<15
IL-2	<5	<17
IL-6	<5	<15
IL-7	<5	<15
IL-8	<5	<15
IL-10	<5	<15
IL-15	<5	<17
IL-18	<5	<15
MCP-1	<5	<15
TNFα	<5	<15

### 3.7 Data management and analysis

#### 3.7.1 Data management

Paper data capture sheets were physically completed for each animal daily. The data was copied into data capture sheets set up in Microsoft Excel and were stored on the primary investigator's laptop, a back-up was copied onto a flash drive, another back-up was stored online in a shared Google drive for all co-investigators to have access to daily. All physical documentation was scanned and stored on the Google drive.

### 3.8 Statistical analysis

For the analysis, variables that are known or suspected to be non-normally distributed, i.e., right-skewed, were log-transformed; these were parasitemia, the leukocyte counts, CRP, GM-CSF, IFNγ, KC-like, all the interleukins, MCP-1 and TNFα. The other variables were not transformed. Variables were then compared between the HD group and the LD group at each time point as well as between each time point and the mean baseline value within each group using linear mixed models, with animal ID as random effect and the Bonferroni adjustment for multiple comparisons. Linear mixed models were used to compare the continuous and ordinal variables. Pairwise correlations between variables were assessed using Spearman's rank correlation. Significance was assessed at P<0.05. Statistical analysis was done using Stata 15 (StataCorp, College Station, TX, U.S.A.).

## Ethics Statements

The experimental study complied with the ARRIVE guidelines and was carried out in accordance he National Institutes of Health guide for the care and use of laboratory animals (NIH Publications No. 8023, revised 1978). Only neutered male beagle dogs were used for the experimental infection to eliminate the effect of oestrus may have had on the experimental outcome. The ethics governing body was the University of Pretoria, Faculty of Veterinary Science Animal Ethics Committee, project number REC048-19.

## CRediT Author Statement

**Brogan Kim Atkinson:** Conceptualization, Data curation, Formal analysis, Investigation, Methodology, Project administration, Visualization, Writing – original draft preparation, Writing – review & editing; **Peter Thompson:** Data curation, Formal analysis, Methodology, Writing – review & editing; **Estee Van Zyl:** Data curation, Investigation, Writing – review & editing; **Amelia Goddard:** Conceptualization, Investigation, Methodology, Writing – review & editing; **Yolandi Rautenbach:** Investigation, Writing – review & editing; **Johan Petrus Schoeman:** Conceptualization, Investigation, Methodology, Funding acquisition, Writing – review & editing; **Varaidzo Mukorera:** Supervision, Writing – review & editing; **Andrew Leisewitz:** Conceptualization, Data curation, Formal analysis, Investigation, Methodology, Project administration, Visualization, Supervision, Funding acquisition, Writing – review & editing.

## Declaration of Competing Interest

The authors declare that they have no known competing financial interests or personal relationships that could have appeared to influence the work reported in this paper.

## Data Availability

Experimental Babesia rossi infection data (Original Data) (Mendeley Data).
